# Adipokines as predictive factor of cardiac function in pediatric patients with chronic kidney disease

**DOI:** 10.3389/fendo.2023.1120445

**Published:** 2023-03-09

**Authors:** Miguel Angel Villasis-Keever, Jessie Nallely Zurita-Cruz, Claudia Zepeda-Martinez, Gabriela Alegria-Torres, Juana Serret-Montoya, Maria de Jesus Estrada-Loza, Beatriz Carolina Hernández-Hernández, Sara Alonso-Flores, Monica Zavala-Serret

**Affiliations:** ^1^ Research Unit in Analysis and Synthesis of the Evidence, National Medical Center XXI Century, Instituto Mexicano del Seguro Social, Mexico City, Mexico; ^2^ Hospital Infantil de Mexico Federico Gómez, Facultad de Medicina Universidad Nacional Autónoma de Mexico, Mexico City, Mexico; ^3^ Department of Pediatric Nephology, Children’s Hospital, National Medical Center XXI Century, Instituto Mexicano del Seguro Social, Mexico City, Mexico; ^4^ Adolescent Medicine Service, Hospital Infantil de Mexico Federico Gómez, Ministry of Health, Secretaria de Salud (SSA), Mexico City, Mexico; ^5^ Department of Pediatric Cardiology, Children’s Hospital, National Medical Center XXI Century, Instituto Mexicano del Seguro Social, Mexico City, Mexico

**Keywords:** chronic kidney disease, leptin, pediatric, free leptin, cardiac function

## Abstract

**Background:**

Adipokines are associated with cardiovascular disease; in chronic kidney disease (CKD) patients adipokines could be useful prognostic factors.

**Objectives:**

To explore whether leptin and adiponectin in kidney replacement therapy (KRT) children could have a role on their cardiac function, in the long-term.

**Design:**

Prospective cohort study was performed with pediatric KRT patients, aged 8 to 17 years who were undergoing hemodialysis or peritoneal dialysis. At enrollment, lipid profile, adipokines (leptin, leptin receptor, free leptin, and adiponectin), anthropometric measurements and cardiological evaluation were determined. At two-year follow-up, a new cardiological evaluation was performed. *Statistical analysis*: Quantitative data are presented as median and interquartile range (IQR). Mann-Whitney U test and Chi-squared were used for the between-group comparison. Multivariate analyzes were performed to determine the association of adipokines levels with ventricular ejection fraction (LEVF).

**Results:**

We included 56 patients, with a median age of 12.5 years. In the first cardiological evaluation, median LVEF was 70.0% (IQR 61%, 76%), 20 patients (35.7%) had some cardiovascular condition, and 10 (17.8%) altered LVEF. At 24-month follow-up, the median LVEF was 70.5% (IQR 65.1%, 77%), while the delta-LVEF values was 3% (IQR -6.5%, 7%). Delta-LVEF were correlated with baseline adipokines serum levels, and the only positive correlation found was with free leptin (r=0.303, p=0.025). In multivariate analysis, levels of free leptin (Coef. 0.12, p<0.036) and leptin (coef. 1.72, p=0.049), as well as baseline LVEF (Coef. -0.65, p<0.001) were associated with delta-LVEF.

**Conclusions:**

Free leptin, leptin and LVEF at the beginning of follow-up were associated with the LVEF decrease at the 24-month follow-up in KRT children.

## Introduction

In recent years, both the prevalence and incidence of chronic kidney disease (CKD) in children have increased (1). Unlike adult patients, the most common causes of CKD in children are congenital malformations; but the increase in overweight/obesity in children may be a contributing factor as well (2).

In adult patients who had CKD during childhood, cardiovascular disease (CVD) is the leading cause of death, with estimates ranging from 23 to 60%. It seems that CVD begins early in CKD children, and arterial hypertension in CKD patients can increase kidney disease progression, due to intraglomerular hypertension, hyperfiltration and increased protein excretion (3, 4). During end-stage CKD, the exhausted adaptive mechanisms and side effects of renal replacement therapy lead to progressive heart failure and accelerated calcification (5).

Adipose tissue is considered an endocrine organ that produces multiple adipocytokines; leptin and adiponectin stand out because they have been identified as mediators of inflammation and may be important markers of chronic systemic inflammation (6, 7). Leptin is a peptide hormone produced by adipocytes, and its serum levels are proportionally correlated to body fat stores (8). As well, leptin exhibits proinflammatory actions, including upregulating the phagocytic function of macrophages, increasing the production of proinflammatory cytokines, and stimulating reactive oxygen species (9). In contrast, adiponectin is produced by the mitochondria of adipocytes, and acts as an anti-inflammatory factor, inhibiting the production of proinflammatory cytokines (10). However, high adiponectin concentrations have been associated with adverse cardiovascular outcomes in adult patients with ischemic heart disease, chronic heart failure and CKD, which has been called the “adiponectin paradox” (11–13).

In developing countries, such as Mexico, kidney transplantation is performed at a lower rate than in developed countries, and several years may pass before a pediatric patient with end-stage renal disease undergoes kidney transplantation. Therefore, it is important to maintain optimal cardiometabolic conditions in the long term in these patients. Leptin and adiponectin have been considered as prognostic factors for the progression of cardiometabolic disorders in patients with overweight/obesity, but information is lacking or controversial in CKD patients. This study aims to explore whether leptin and adiponectin in kidney replacement therapy (KRT) children could have a role on their cardiac function, in the long-term.

## Methods

### Subjects

A prospective cohort study was carried out from January 2018 to December 2020 at two tertiary pediatric care centers in Mexico City: Hospital de Pediatría (Mexican Institute of Social Security) and Hospital Infantil de México Federico Gómez (Mexico Ministry of Health). In both centers, all pediatric KRT patients are usually cared for by a multidisciplinary team that includes pediatric nephrologists, pediatric endocrinologists, pediatric cardiologist, psychologists, and nutritionists.

Children aged between 8 and 17 years with stage V CKD according to the Kidney Disease: Improving Global Outcomes (KDIGO) staging scale (14), and who were receiving peritoneal dialysis or hemodialysis were considered eligible to participate in the study. Patients who were scheduled for kidney transplantation in the next 12 months, with diagnosed with diabetes mellitus, those did not agree to participate, or those who had incomplete clinical and biochemical evaluation data were excluded. The cohort follow-up duration was 24 months. All included patients were selected using a consecutive sampling technique.

According to the Declaration of Helsinki, the protocol was approved by hospitals’ ethics and research committees, under registry numbers: R-2018-3603-075 & HIM-2017-117. A parent or legal guardian signed an informed consent form, and each child provided written assent.

### Anthropometry

The anthropometric indicators of each patient were recorded by a certified nutritionist. Height was measured to the nearest 0.1 cm with a SECA model 769 stadiometer (SECA 769, SECA Corp. Oakland Center Columbia, MD, USA). Weight and body fat percentage measurements were conducted using the bioimpedance method (Tanita BC-568 Segmental Body Composition Monitor, Tokyo, Japan) with the patients barefoot and wearing only underwear. Anthropometric measurements were performed both, at the beginning and at the end of the 24-month follow-up.

### Serum hormones and biochemistry level measurements

Blood samples were obtained from the forearm of each subject *via* the antecubital vein, between 7:00 and 8:00 a.m. after a minimum of 12 hours of fasting during the baseline visit. Serum aliquots were separated (centrifuged at 4°C; 3000 rpm; 15 min) and frozen at -80°C until biochemical analysis. Leptin and leptin receptor levels were measured using an enzyme-linked immunosorbent assay (ELISA) (Human Leptin Duo Set, DY 398, Human Leptin Receptor, CAT DY 389, R&D Systems, Minneapolis, MN, USA); Human Adiponectin DuoSet (DY1065), R&D Systems, Minneapolis, MN, USA). Plates were read using an ELISA microplate reader (Labsystems Multiskan EX, MTX Labsystems Inc., Vienna, VA) and were determined in duplicate according to the manufacturer’s instructions. The plates were assessed using an ELISA microplate reader (Labsystems Multiskan EX, MTX Labsystems Inc., Vienna, VA) and were assessed in duplicate as per the manufacturer’s instructions. Intra- and interassay coefficients of variation <7% were considered acceptable. A standard curve was also generated for each assay. Free leptin levels were calculated by dividing the levels of total leptin by that of leptin receptors (15). Creatinine and urea levels were determined by colorimetric enzymatic methods (Bayer Diagnostics, Puteaux, France). All electrochemiluminescence immunoassays (ECLIAs) were performed using a COBAS 6000 e601 (Roche Diagnostics GmbH, Indianapolis, IN, USA) in duplicate according to the manufacturer’s recommendations. Intra- and interassay coefficients of variation < 7% were considered acceptable. A standard curve was also generated for each assay.

### Cardiology evaluation

Cardiological evaluation was performed by a certified pediatric cardiologist, at baseline and at 24-month follow-up. All patients underwent to a physical examination, chest X-ray, electrocardiogram, as well as echocardiographic evaluation. The latter was performed with Philips iE33 cardiovascular ultrasound machine with xMATRIX 5 MHz, using Pediatric xMATRIX X 2-7 MHz transducers.

### Definitions

Patients with BMI <5^th^ percentile were considered malnourished, obesity with BMI > 95^th^ percentile, and overweight with BMI > 85^th^ percentile, according to the 2000 CDC Growth Charts (16). Patients with <2 standard deviations of height for age, BMI was calculated considering the age that corresponds to the 50^th^ percentile of actual height.

Hemodialysis and peritoneal dialysis treatment adequacy was calculated by Kt/V (K, dialyzer clearance of urea; t, dialysis time; and V, volume of distribution of urea). In hemodialysis patients, Kt/V > 1.2/week was considered adequate; in the case of peritoneal dialysis, when Kt/V > 1.8/week (17, 18).

There were two criteria for hypertension according to age: in patients < 13 years, when systolic or diastolic blood pressure was ≥95^th^ percentile for age, height, and sex. While for those > 13 years-old, when systolic blood pressure was ≥130 mmHg, or diastolic blood pressure ≥80 mmHg (3).

Based on the cardiology evaluation, patients with hypertensive cardiomyopathy, dilated cardiomyopathy, aortic valve dysfunction were identified. Patients considered to have altered left ventricular ejection fraction (LVEF) had values <40%, as well as those with LVEF >40% but who also had clinical data of heart failure (19).

### Statistical analyses

Quantitative data are presented with median and interquartile range (IQR) since they did not show normal distribution, according to Shapiro-Wilk test. LVEF delta was calculated by the difference in the LVEF value at the end of follow-up, minus the baseline value, of each patient.

Two groups were formed to carry out the different analyses: with and without altered LVEF; Mann-Whitney U test and Chi-squared were used for the between-group comparison. Baseline cytokine levels were correlated with delta-LVEF values using Pearson’s correlation coefficient. Two models of lineal regression analysis were performed to determine the association between basal cytokines levels with delta-LVEF values, adjusted for nutritional status (overweight/obesity), hypertensive cardiomyopathy, hemodialysis and time on renal replacement therapy.

A p-value < 0.05 was considered statistically significant. All analyzes were performed with STATA v.11.0.

## Results

**Table 1** shows the baseline characteristics of the 56 included patients, noting that 10 patients (17.8%) already had altered LVEF. There were patients from 10 to 14 years-old, with similar sex ratio. The majority had a normal nutritional status (57.1%), and 16 patients (27.9%) were overweight or obese. Regarding the CKD etiology, the most frequent was CAKUT in 46.4% (n=26), followed by glomerulopathy (28.6%, n=16).

**Table 1:** Baseline in CPK pediatric patients’ characteristics, stratified by altered left ventricular ejection fraction ^1^.

Characteristic	Total n = 56	Altered left ventricular ejection fraction		p^2^
		No n = 46	Yes n = 10	
Age, y				
Median (interquartile range)	12.5 (10.5, 14.5)	12 (11, 15)	13 (10, 13)	0.827
**Sex, %**				
Female	28 (50.0)	24 (52.2)	4 (40.0)	0.364
Male	28 (50.0)	22 (47.8)	6 (60.0)	
Somatometry, median (interquartile range)				
Weight, kg	35.7 (27.5, 41.2)	35.5 (25.4, 41.3)	40.5 (23.8, 41.1)	0.830
Height, cm	143 (130, 153)	144 (130, 152)	140 (128, 154)	0.089
Height z score	-1.9 (-3.3, -0.9)	-1.5 (-3.2, -0.9)	-3.6 (-4.1, -1.8)	**0.014**
Body mass index, kg/m^2^	17.4 (16.0, 20.0)	17.3 (15.7, 19.1)	22.9 (18.5, 23.7)	**0.035**
Body mass index z score	-0.06 (-1.09, 1.2)	-0.15 (-1.1, 1.1)	0.09 (-0.9, 2.0)	0.466
Nutritional status, %				
Normal	32 (57.1)	28 (60.9)	6 (60.0)	0.138
Malnutrition	8 (14.3)	6 (13.0)	0 (0.0)	
Overweight	10 (17.9)	9(19.6)	0 (10.0)	
Obesity	6 (10.7)	3 (6.5)	4 (30.0)	
Etiology of chronic kidney disease				
CAKUT	26 (46.4)	20.0 (43.5)	6 (60.0)	0.794
Glomerulopathy	16 (28.6)	14 (30.4)	2 (20.0)	
immunological	4 (7.1)	4 (8.7)	0 (0.0)	
Indeterminate	10 (17.9)	8 (17.4)	2 (20.0)	
Replacement treatment; %				
Hemodialysis	12 (21.4)	8 (17.4)	4 (40.0)	0.126
Peritoneal dialysis	44 (78.6)	38 (82.6)	6 (60.0)	
Age at diagnosis of CKD, y				
Median (interquartile range)	9.0 (5.5, 12.0)	10.0 (4.0, 12.0)	9.0 (7.0, 9.0)	0.575
Time of renal replacement, months				
Median (interquartile range)	18.0 (7.0, 31.0)	18.0 (7.0, 31.0)	15.0 (10.0, 24.0)	0.574
Hypertension, %				
Presence	34 (60.7)	28 (60.8)	6 (60.0)	0.613
Kt/V, l/week				
Median (interquartile range)	1.88 (1.12, 3.40)	2.12 (1.51, 3.42)	1.80 (1.11, 2.65)	0.751
Altered	15 (26.8)	13 (28.3)	2 (20.0)	0.461
Left ventricular ejection fraction, %				
median (interquartile range)	70 (61, 76)	72 (61, 80)	62 (61, 64)	**0.001**
Cardiological assessment, %				
Normal	36 (64.3)	32 (69.7)	4 (40.0)	0.124
Hypertensive cardiomyopathy	14 (25.0)	10 (21.7)	4 (40.0)	
Valve dysfunction	4 (7.1)	2 (4.3)	2 (20.0)	
Dilated cardiomyopathy	2 (3.6)	2 (4.5)	0 (0.0)	
Adipokines, median (interquartile range)				
Leptin, ng/ml	4.6 (3.0, 6.2)	3.8 (2.1, 6.1)	6.0 (5.3, 6.6)	**0.020**
Leptin receptor, ng/ml	0.5 (0.1, 2.2)	0.4 (0.1, 1.9)	1.3 (0.1, 4.4)	0.314
Free leptin, ng/ml	6.4 (1.1, 60.5)	6.6 (1.2, 59.8)	5.1 (0.7, 60.5)	0.789
Adiponectin, µg/ml	6.1 (5.3, 6.3)	6.0 (5.3, 6.2)	6.4 (6.3, 6.4)	**0.018**

^1^Values are n (%) or median (interquartile range). ^2^Statistics was conducted with χ^2^ or Mann-Whitney U test as appropriate. Bold values are statistical significance.

Although there were more peritoneal dialysis patients in the normal LVEF group (82.6% vs 60%), the difference was not statistically significant. Both the time on renal replacement therapy, as well as Kt/V and frequency of hypertension were similar between the two groups (**Table 1**).

Regarding cardiology evaluation, at baseline the median LVEF was 70% (IQR 61% to 76%) in the 56 patients. As expected, LVEF was statistically lower in the altered LVEF group compared to the other group (62% vs 72%), p=0.001 As also shown in **Table 1**, in the altered LVEF group the proportion of patients with hypertensive cardiomyopathy and valvular dysfunction was higher than the other group, which was not statistically significant.

Cytokine levels were different between the two groups. Compared with the normal LVEF group, leptin (p=0.02), leptin receptor (p=0.31), and adiponectin (p=0.018) levels were higher in the altered LVEF group, whereas free leptin levels were lower (p=0.78). Furthermore, cytokine levels were compared according to nutritional status; as shown in **Table 2**, leptin (6.5 ng/ml *vs* 2.1 ng/ml, p=0.002) and free leptin (65.1 ng/ml *vs* 0.6 ng/ml, p=0.028) were higher in patients with obesity compared to those with malnutrition (**Table 1**).

**Table 2:** Baseline adipokines serum levels, stratified by nutritional status (n=56) ^1^.

	*Nutritional status*		Overweight n=10	Obesity n=6	p^2^
	Normal n=32	Malnutrition n=8			
Adipokines, median (interquartile range)					
Leptin, ng/ml	5.0 (3.5, 6.1)	2.1 (0.9, 3.3)	4.1 (1.8, 6.2)	6.5 (5.3, 6.5)	**0.002**
Leptin receptor, ng/ml	0.4 (0.1, 1.7)	2.3 (1.9, 3.4)	0.36 (0.1, 1.9)	0.1 (0.1, 47.9)	0.093
Free leptin, ng/ml	9.5 (2.7, 59.8)	0.6 (0.4, 1.7)	5.9 (1.6, 60.5)	65.1 (0.1, 65.9)	**0.028**
Adiponectin, µg/ml	6.1 (5.6, 6.3)	5.9 (4.1, 6.3)	5.8 (4.8, 6.1)	6.4 (6.0, 6.9)	0.073

^1^Values are median (interquartile range). ^2^Statistics was conducted with Kruskal Wallis test. Bold values are statistical significance.

When analyzing the levels of adipokines with the baseline patients’ characteristics through logistic regression, leptin levels (OR 3.11; 95%IC 1.06, 9.11, p=0.038), leptin receptor levels (OR 1.06; 95%IC 1.001, 1.12, p=0.043) hypertensive cardiomyopathy (OR 18.37; 95% IC 1.28, 263.1, p=0.032) were associated with altered LVEF, contrary to adiponectin levels (OR 1.22; 95% IC 0.67, 2.22, p=0.503).

### End of follow-up

**Figure 1:**
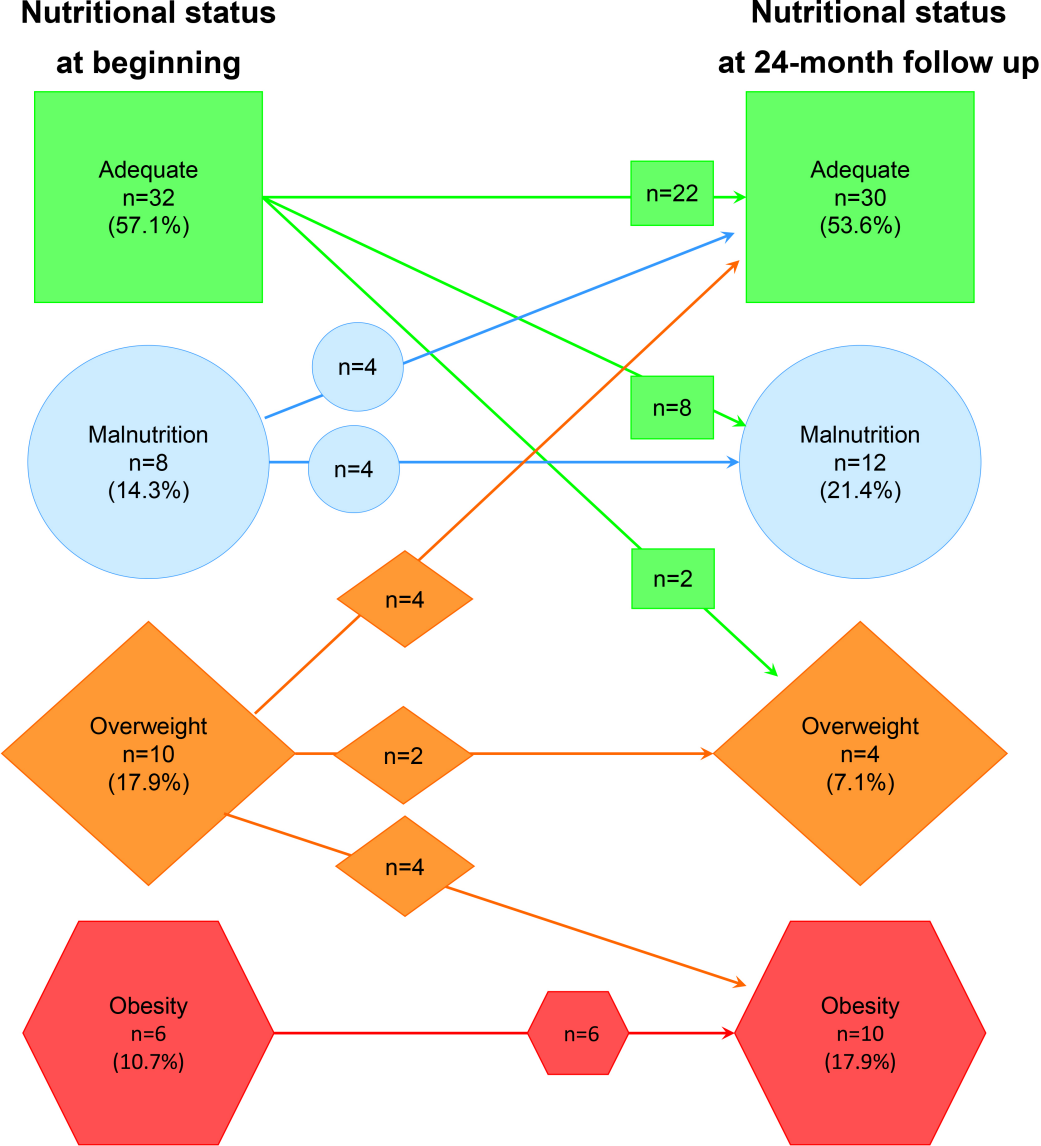
Change in nutritional status from baseline to 24 months of follow-up in chronic kidney disease pediatric patients.

After 24-month follow-up, no change was observed in BMI z-score (median -0.06 *vs* median -0.18, p=0.99), in the 56 patients. But the proportion of patients with malnutrition (21.4%) and obesity (17.8%) increased (**Figure 1**). As cardiac function at the end of follow-up, LVEF median was 70.5% (IQR 65.1%, 77%), while the delta-LVEF was 3% (IQR -6.5%, 7%).

**Figure 2:**
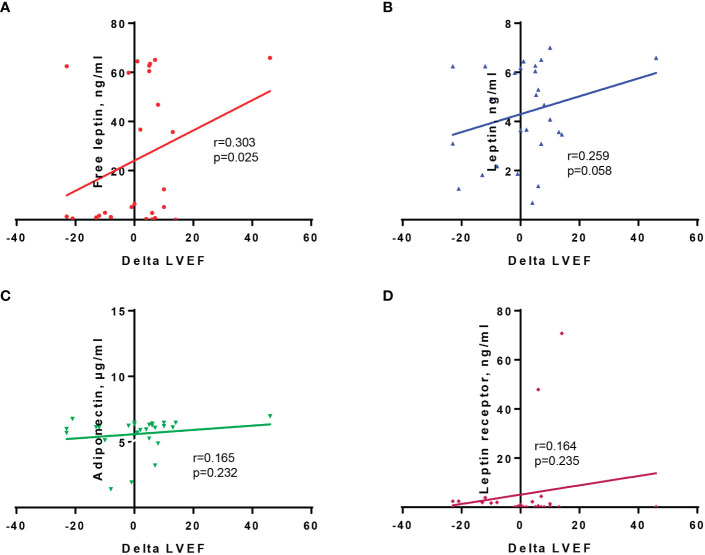
Correlation between delta-LVEF values and baseline serum levels of free leptin **(A)**, leptin **(B)**, adiponectin **(C)**, and leptin receptor **(D)**.

**Figure 2** presents the correlation analyses between baseline cytokine levels and delta-LVEF values. As shown, leptin (r=0.259, p=0.058) and free leptin levels (r=0.303, p=0.025) were positively correlated with delta-LVEF. This was not observed for serum adiponectin levels (r=0.165, p=0.232).

Finally, according to the linear regression analyses, in the first model free leptin levels (coef. 0.12; 95%IC 0.08, 0.24, p=0.036) and baseline LVEF values (coef. -0.65; 95%IC -0.91, -0.38, p<0.001) were associated with delta-LEVF, while in the second model leptin levels (coef. 1.72; 95%IC 0.01, 3.44, p=0.049) and baseline LVEF (coef. -0.63; 95%IC -0.90, -0.35, p<0.001) were associated with delta-LEVF, **Tables 3A**, **B**, respectively.

**Table 3A:** Linear regression analysis to identify the association of free leptin with delta-left ventricular ejection fraction at 24-month follow-up in CPK pediatric patients (n=56).

	Coeficient	CI 95%	p
**Free leptin, ng/ml**	0.10	0.001, 0.22	**0.049**
**Adiponectin, µg/ml**	1.28	-0.96, 3.25	0.220
**Hypertensive cardiomyopathy**	-2.40	-6.7, 4.37	0.472
**Baseline left ventricular ejection fraction**	-0.65	-0.92, -0.38	**<0.001**
**Overweight or obesity at baseline**	5.84	-0.56, 12.24	0.073
**Replacement treatment with hemodialysis**	0.94	-2.82, 4.71	0.616
**Time of renal replacement, months**	0.11	-0.06,0.29	0.214
**Modification of nutrition status**	-4.67	-11.4, 2.10	0.172

IC, confidence interval; BMI, Body mass index. Bold values are statistical significance.

**Table 3B:** Linear regression analysis to identify the association of leptin and leptin receptor with delta-left ventricular ejection fraction at 24-month follow-up in in CPK pediatric patients (n=56).

	Coeficient	CI 95%	p
**Leptin, ng/ml**	1.72	0.01, 3.44	**0.049**
**Leptin receptor, ng/ml**	0.09	-0.09, 0.29	0.302
**Adiponectin, µg/ml**	1.90	-0.33, 4.14	0.094
**Hypertensive cardiomyopathy**	-3.30	-10.4, 3.72	0.345
**Baseline left ventricular ejection fraction**	-0.63	-0.90, -0.35	**<0.001**
**Overweight or obesity at baseline**	5.92	-0.47, 12.31	0.069
**Replacement treatment with hemodialysis**	0.31	-3.42, 4.05	0.868
**Time of renal replacement, months**	0.11	-0.05,0.28	0.167
**Modification of nutrition status**	-5.87	-12.8, 1.14	0.099

IC, confidence interval; BMI, Body mass index. Bold values are statistical significance.

## Discussion

To our knowledge, this is the first study where leptin and adiponectin levels have been evaluated as potential prognostic markers of cardiac function in CKD pediatric on renal replacement therapy. Our results seem to indicate that both elevated serum leptin and free leptin levels are associated with a decrease in LVEF, at two years of follow-up. This information could be relevant, since the most common cause of mortality among CKD patients is cardiovascular disease.

Cardiovascular diseases in pediatric patients can be present in CKD early stages. In our study, it was identified in about a third of the 56 included patients, mainly due to hypertensive cardiomyopathy. According to Groothoff et al, they reported 61.5% of cardiac abnormalities in a cohort of 140 pediatric patients followed for 20 years, since 1972. This high frequency is probably related to the time of the study, since kidney transplantation was not performed in a timely manner as it is today, therefore the time in renal replacement therapy was longer (20). Moustafa et al. reported 88% of cardiac alterations due to left ventricular hypertrophy and left ventricular dilatation, but the frequency of hypertension was higher (72%) than in our study (60.7%) (20, 21).

In recent years, it has been described that overweight and obesity can cause cardiovascular disorders in CKD patients, which could aggravate the damage caused by renal failure (22). However, the effect that adipocytokines may have in CKD children is unknown, particularly on heart function.

Adipokines change according to nutritional status; for example, leptin is a good indicator of the amount of adipose tissue in the body (8). As we observed in this study, patients with overweight or obesity had the highest serum leptin and free leptin levels, while malnourished patients had the lowest concentrations (23, 24). In patients only with obesity, elevated serum leptin concentrations lead to increase cardiovascular risk (9); however, in the context of chronic diseases patients, such as CKD, this situation is not clear. CKD patients could be at greater risk of malnutrition and inflammatory-related diseases due to the release of inflammatory cytokine by adipocytes, and the involvement of regulatory molecules, as myostatin, hepatocyte growth factor and soluble Toll-like receptor 4 (25). Sarcopenia as a chronic proinflammatory state increases the risk of damage to target organ, such as impaired cardiac function (26–28).

We observed that the decrease in delta-LVEF was associated with free leptin levels. This finding is consistent with studies conducted in patients with anorexia, in whom the energy balance is negative because of insufficient caloric intake. In these patients, increased leptin receptor levels may represent a protective mechanism that decreases the bioavailability of free leptin that would further conserve energy (29, 30). Adult patients on hemodialysis and sarcopenia have a worse prognosis for cardiovascular events and mortality, which has been related to low fat content, as a consequence of the proinflammatory state that occurs in sarcopenia (26–28). As well, it has also been reported that high levels of angiotensin II are negatively associated with skeletal muscle strength (31). Angiotensin II acts on IGF-I/insulin signaling pathways, by decreasing Akt phosphorylation and activating muscle proteolysis by the ubiquitinproteasome system (32) and caspase-3 apoptotic pathways in muscle (31, 33). Thus, a mechanism by which angiotensin II induces muscle atrophy is by disrupting the IGF-I system.

Similar to our study, increased adiponectin levels have also been observed in adult patients with heart failure, diabetes mellitus, and CKD. The high levels of circulating adiponectin could be attributed to the counterregulatory upregulation of adiponectin production in response to stress caused by severe chronic diseases (12, 13, 34, 35). Adiponectin has multiple beneficial phenotypic expression effects that include anti-inflammatory, antiatherogenic or cardioprotective actions (36–38). Therefore, it is likely that the high levels of circulating adiponectin in these patients can be partially explained by the compensatory upregulation of adiponectin production in response to severe chronic stress related to CKD. Furthermore, in patients with heart failure downregulation of adiponectin receptor is associated with decreased downstream signaling, such as inactivation of the PPAR-α/AMPK pathway, and downregulation of several target genes in skeletal muscles, resulting in functional resistance to adiponectin (39, 40). However, more studies are needed to explain the mechanism of this compensatory adiponectin response in cardiovascular diseases.

On the other hand, myocardial remodeling secondary to hypertension is mainly due to hypertrophy of cardiomyocytes, interstitial fibrosis, and alterations in the wall of the intramyocardial arteries. This is an adaptive response to overload as an attempt to normalize systolic stress, which alters the left ventricle global function (41). Matteucci et al. reported a regression of left ventricular hypertrophy and improvement of left ventricular systolic function when blood pressure is controlled (42). Persistent uremia results in thickening of myocardial cells and concentric remodeling of the left ventricle together with activation of the intracardiac renin-angiotensin system, which induces hyperaldosteronemia. This promotes cardiac fibrosis *via* signals that induce production of profibrotic growth factors, which causes myocardial remodeling. Late renal transplantation causes a longer exposure to uremia, which increase in the probability of developing hypertensive cardiomyopathy, as we observed in our patients (4, 5).

Finally, we must recognize the limitations of the study, mainly due to the small sample size, which may affect the interpretation of multivariate analyses. Therefore, more studies should be carried out to verify whether adipokines can be considered as prognostic markers for the deterioration of cardiac function in CKD pediatric patients. In these studies, it seems appropriate to include measurements of adipokines and body composition at the end of follow-up.

## Conclusions

Baseline leptin, free leptin levels and LVEF were associated with the decrease of LVEF at the 24-month follow-up in CKD pediatric patients.

## Data availability statement

The original contributions presented in the study are included in the article/Supplementary Material. Further inquiries can be directed to the corresponding author.

## Ethics statement

According to the Declaration of Helsinki, the protocol was evaluated and approved by the ethics and research committee of the hospital under registry number R-2018-3603-075 & HIM-2017-117. A parent or legal guardian signed an informed consent form, and each child provided written assent according to the recommendations of the Declaration of Helsinki. Written informed consent to participate in this study was provided by the participants’ legal guardian/next of kin.

## Author contributions

Conceptualization Methodology & Formal analysis: MV-K and JZ-C; Investigation: JZ-C, CZ-M, GA-T, JS-M, ME-L, BH-H, SA-F, and MZ-S; Writing, review & editing: MV-K and JZ-C. All authors contributed to the article and approved the submitted version.
